# Organic fibrous nanostructures *via* the droplet-assisted growth and shaping (DAGS) mechanism

**DOI:** 10.1039/d5ra01919b

**Published:** 2025-07-04

**Authors:** Rabab Azizi, Stefan Seeger

**Affiliations:** a Department of Chemistry, University of Zurich Winterthurerstrasse 190 8057 Zurich Switzerland s.seeger@uzh.ch

## Abstract

The Droplet-Assisted Growth and Shaping mechanism (DAGS) represents a bottom-up approach for the fabrication of versatile one-dimensional polymeric (1D) nanomaterials and involves the polymerisation of a highly reactive monomer with water nanodroplets formed on a substrate surface. The unidimensional growth of the resulting polymer is sustained with its water insolubility. To date, all 1D polymeric nanostructures grown *via* the DAGS mechanism were either based on silicone, alumina, or germanium oxide but not on a carbonic backbone. In this paper, we demonstrate for the first time that the DAGS mechanism can also be used for the growth of organic 1D polymeric nanostructures using ethyl-2-cyanoacrylate (ECA) as a monomer. The polymerisation is carried out in *n*-hexane/toluene mixtures with different water contents (WCs). The obtained poly(ethyl-2-cyanoacrylate) (PECA) fibrous nanostructures (PECA-FNS), which were coated on glass, manifested as nanofibers and nanoribbons with an aspect ratio ranging from 4.9 to 18.3. Attenuated total reflectance infrared (ATR-IR) spectroscopy revealed the presence of the carbonyl bond on the coated glass substrates, confirming the presence of the PECA-FNS. The topography and the root mean square roughness (Sq) of the PECA-FNS were examined *via* atomic force microscopy (AFM). Both static contact angle measurements and UV-Vis spectrophotometry showed that the PECA-FNS coatings displayed a high transparency and moderate hydrophobicity.

## Abstract 2

This study unveils the formation of organic fibrous nanostructures *via* the DAGS mechanism, achieved by polymerising ECA in solution with water, demonstrating a novel approach to synthesise versatile 1D nanomaterials.

## Graphical abstract

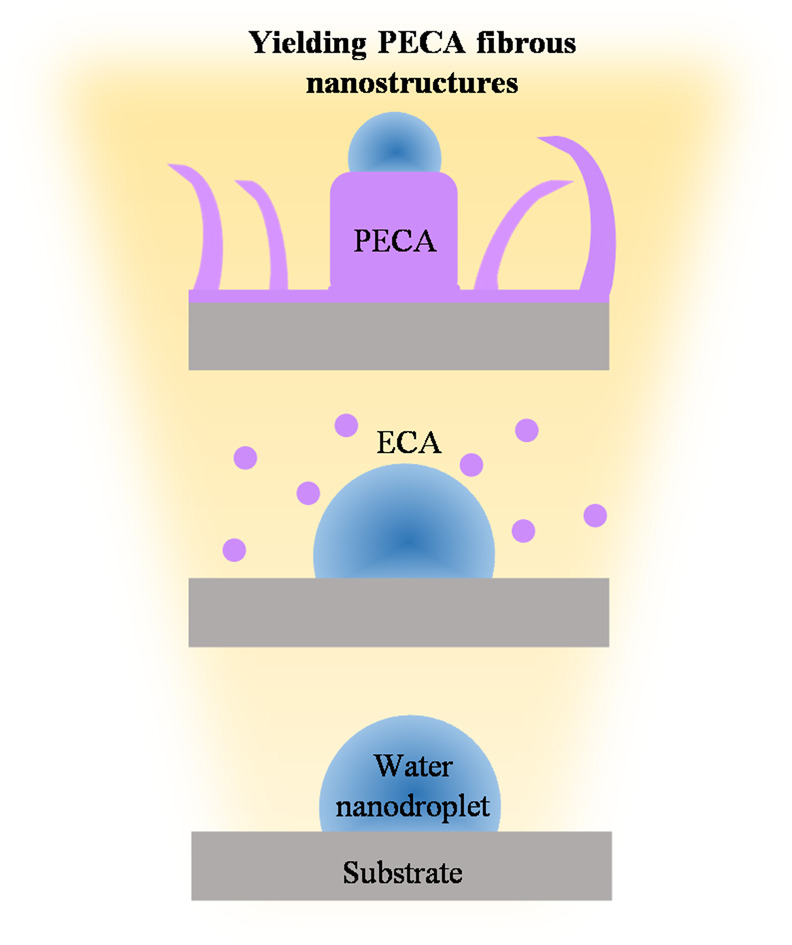

## Introduction

One-dimensional (1D) polymeric nanostructures such as nanofibers, nanotubes, and nanowires, are nanomaterials with a typical diameter ranging from 1 to 100 nm.^1,2^ Compared to their bulk polymeric analogues, the miniaturisation into the nanoscale usually results in novel chemical, thermal, and mechanical properties,^3^ for example, the high surface-to-volume ratio and optical anisotropy exhibited by nanofibers.^4^ Among the various methods for the fabrication of 1D polymeric nanostructures, techniques such as electrospinning, self-assembly, and templating are widely employed.^2,5,6^ However, electrospinning and self-assembly processes, while very useful for creating 1D nanostructures, often face challenges in scalability and precise control of the morphology.^7,8^

A few decades ago, Artus *et al.* introduced the droplet-assisted growth and shaping (DAGS) mechanism, a bottom-up approach for manufacturing 1D polymeric nanostructures. This method uses water nanodroplets formed on a substrate (*e.g.* glass) as confined reaction volumes. The water droplets act as initiation centres of reactive inorganic monomers, such as silanes, and the unidimensional growth of the resulting polymer is ensured by its water-insoluble nature.^9^ The advantages of the DAGS mechanism include mild reaction conditions, *i.e.* processing at room temperature and atmospheric pressure, as well as the ability to achieve one-dimensional growth of the polymers in both gas and/or liquid phase.^9,10^ For instance, the straightforward implementation of the DAGS mechanism makes it suitable for scaling up the production of superhydrophobic coatings at the pilot plant level.^11^ Moreover, it has been successfully applied to synthesise further various inorganic 1D polymers, including silicone nanofilaments, nanorods, helices, germanium oxide nanofilaments, and alumina necklaces.^9,12,13^ Our present strategic vision entails not only the growth of diverse inorganic but also organic and eventually hybrid 1D polymeric nanostructures, which are particularly well-known for their tuneable and synergistic properties.^14^ Therefore, the main aim of this paper is to demonstrate the universality of the DAGS mechanism and to validate its applicability to organic monomers. For this purpose, we have chosen ethyl-2-cyanoacrylate (ECA), an organic monomer, mostly commercialised as an instant adhesive.^15^ ECA undergoes a rapid anionic polymerisation in the presence of trace amounts of water^16,17^ and was previously used to obtain nanofibers in the gas phase at high relative humidity values.^18^ Although these PECA nanofibers were obtained only by the mean of specific initiators such as alcohols or salts such as NaCl, KCl, CH_3_COOK,^19–21^ while our unique method relies solely on the initiation of water nanodroplets present on the substrate surface. Namely, the ECA anionic polymerisation in our work was carried out in *n*-hexane/toluene mixtures with water contents ranging from 30 to 120 ppm using pre-treated glass slides as substrates. Scanning electron microscope (SEM) images unveiled versatile PECA fibrous nanostructures (PECA-FNS) including nanofibers and nanoribbons, with an aspect ratio up to ∼18.3. This breakthrough represents a new generation of 1D nanostructures, demonstrating the unprecedented successful transition of the DAGS mechanism from inorganic to organic 1D nanomaterials.

## Experimental section

### Materials

Glass slides (25 mm × 60 mm × 1 mm) from Epredia-Menzel were used as a substrate. ECA *n*-hexane and toluene were purchased from Merck, Switzerland. Hydranal Coulomat AG used as a Karl Fischer agent was purchased from Fischer Scientific, Switzerland. Deconex 11 Universal was obtained from Borer Chemie, Switzerland. All solvents and chemicals are of analytical grade and were used as received unless otherwise specified. Milli-Q water was generated from the Simplicity UV system.

### Methods

#### Cleaning and treatment of the glass substrate

Prior to coating with ECA, the glass slides were first submerged in a 10% (vol) Deconex solution and sonicated at 50 °C for 30 minutes and then rinsed with copious amounts of Milli-Q water. Finally, the glass slides were dried using a nitrogen gun at an ambient temperature of 21 ± 1 °C.

#### Anionic polymerisation of ethyl-2-cyanoacrylate in *n*-hexane and its toluene mixtures

**Fig. 1:**
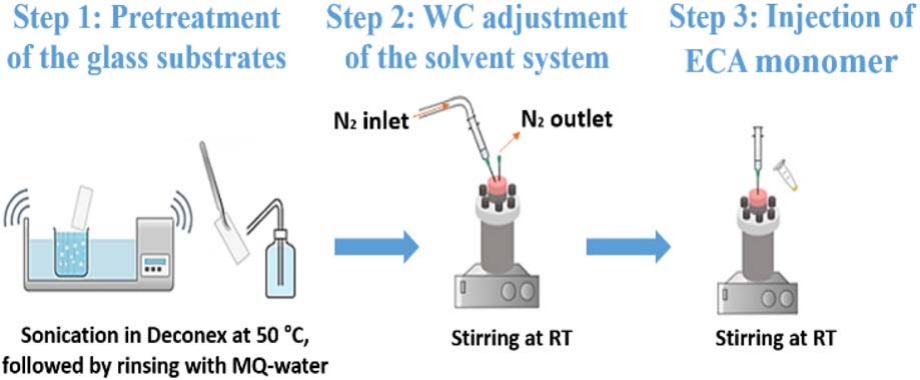
Experimental procedure of the ECA anionic polymerisation in *n*-hexane and its mixtures with toluene (created using Chemix online editor).

The anionic polymerisation of ECA was carried out in *n*-hexane and its toluene mixtures. The volumetric ratios of the two organic solvents *V*_Hex_ : *V*_Tol_ were 4 : 1, 3 : 2, 2 : 3, and 1 : 4, respectively. The water content in each mixture was 30, 50, 90, and 120 ppm with a permissible variation of ±7 ppm. The previously cleaned glass substrates were put vertically in a cylindrical reactor with an inner cavity lined with Teflon to ensure inertness towards organic solvents and avoid the adhesion of monomer on the reactor walls. A total volume of 100 mL of *n*-hexane or *n*-hexane/toluene mixture was introduced into the reactor, which was then tightly sealed. The water content in the organic solvent was adjusted under constant stirring (300 rpm) at a temperature of 21 ± 1 °C using a stream of either dry or wet nitrogen gas. The latter was generated by passing dry nitrogen through a bottle filled with Milli Q-water. The water content was determined using a Mettler Toledo C20 Karl-Fischer coulometer equipped with a platinum reference electrode and a diaphragmaless generator electrode. Once the desired water content is reached, at least two further values are measured for verification. Subsequently, 0.85 mmol of ECA was injected into the reactor and allowed to polymerise for 18 to 22 hour under continuous stirring (120 rpm). The PECA-coated substrates are finally taken out and dried at ambient conditions using a nitrogen gun and preserved in glass slide mailers made from polypropylene until later analysis. The entire experimental procedure for the fabrication of PECA nanostructures is depicted in Fig. 1.

#### Characterisation of PECA nanostructures

The scanning electron microscopy (SEM) images were acquired using a Zeiss Gemini SEM 450 at a voltage of 10 kV and samples were sputter-coated with a 5 nm platinum layer. The diameter and length of the nanofibers were measured manually using ImageJ software. Attenuated total reflectance-Fourier transform infrared (ATR-FTIR) measurements were carried out using a Bruker Vertex 70 (Bruker Optic GmbH, Germany) with a single reflection diamond ATR accessory over a range of 700–3200 cm^−1^ and a resolution of 2 cm^−1^. The topography of the PECA-FNS was measured in tapping mode *via* a MFP-3D AFM from Asylum Research using a HQ: NSC15/Al BS probe from MikroMasch. The scan rate was 0.5 Hz. Open-source Gwyddion software package as well as the Asylum Research build-in software were used to process the AFM data and determine the roughness of the samples. Static contact angle measurements were conducted using a DSA100 contact angle goniometer (Krüss GmbH, Germany). The procedure involved dispensing a 10 μL ultrapure water droplet onto the sample surface through the instrument's integrated needle. The profile of the droplet was then analysed using the Young–Laplace fit method. For statistical reliability, measurements were taken at three distinct locations on each sample. For the transparency test, transmittance of the coated samples with the PECA-FNS as well as of a pretreated glass slide and a glass slide coated with crude PECA was measured between 300 and 800 nm using a PerkinElmer Lambda 950 UV-Vis spectrophotometer (Llantrisant, United Kingdom). Transmittance measurements of the background were performed against air.

## Results and discussion

### ECA anionic polymerisation in *n*-hexane and its toluene mixtures

**Scheme 1:**
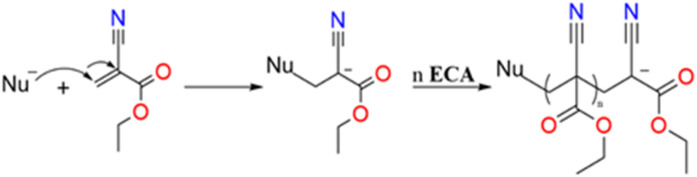
Anionic polymerisation of ECA in the presence of a nucleophile, for example a water molecule.

The polymerisation of ECA in *n*-hexane and its mixtures with toluene proceeds *via* an anionic mechanism, initiated by trace amounts of water. We hypothesised that the rapid reactivity of ECA with even minimal water content would make it a suitable and a promising candidate for extending the DAGS mechanism from inorganic to organic 1D nanostructures structures. The water molecules are both involved in the initiation and termination steps. The initiation step occurs when a water molecule attacks the ECA β-carbon, leading to the formation of a carbanion, which will react with a further ECA molecule. The propagation of the macromolecular chain will continue as long as the monomer is available or as long as there is no chain termination, for example *via* a proton transfer.^22^ The detailed anionic polymerisation mechanism of ECA is shown in Scheme 1.

**Fig. 2:**
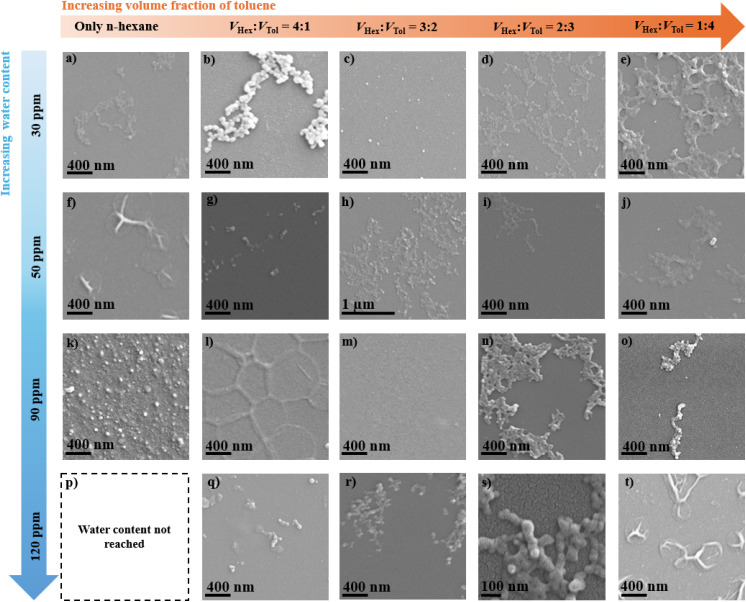
SEM top-view images of the PECA-NS formed during the anionic polymerisation in *n*-hexane/toluene mixtures with different WCs: (a) pure *n*-hexane, (b) *V*_Hex_ : *V*_Tol_ = 4 : 1; (c) *V*_Hex_ : *V*_*T*ol_ = 3 : 2; (d) *V*_Hex_ : *V*_Tol_ = 2 : 3; (e) *V*_Hex_ : *V*_Tol_ = 1 : 4; (a–e) WC = 30 ppm; (f) pure *n*-hexane; (g) *V*_Hex_ : *V*_Tol_ = 4 : 1; (h) *V*_Hex_ : *V*_Tol_ = 3 : 2; (i) *V*_Hex_ : *V*_Tol_ = 2 : 3; (j) *V*_Hex_ : *V*_Tol_ = 1 : 4; (f–j) WC = 50 ppm; (k) pure *n*-hexane; (l) *V*_Hex_ : *V*_Tol_ = 4 : 1; (m) *V*_Hex_ : *V*_Tol_ = 3 : 2; (n) *V*_Hex_ : *V*_Tol_ = 2 : 3; (o) *V*_Hex_ : *V*_Tol_ = 1 : 4; (l–o) WC = 90 ppm; (p) WC not reached in pure *n*-hexane; (q) *V*_Hex_ : *V*_Tol_ = 4 : 1; (r) *V*_Hex_ : *V*_Tol_ = 3 : 2; (s) *V*_Hex_ : *V*_Tol_ = 2 : 3; (t) *V*_Hex_ : *V*_Tol_ = 1 : 4; (l–o) WC = 120 ppm.

We initially investigated the growth of PECA-based coatings on glass, using water contents (WCs) in the range of 30, 50, 90, and 120 ppm in *n*-hexane and *n*-hexane/toluene mixtures with a volumetric ratio *V*_Hex_ : *V*_Tol_ = 4 : 1, 3 : 2, 2 : 3, and 4 : 1. A water content of 120 ppm could not be reached in pure *n*-hexane. Indeed, the maximum reachable water content in pure *n*-hexane was reported to be 90 ppm at 25 °C.^23^ The water content was systematically adjusted to find the optimal amount of initiator (water) for the formation of PECA 1D nanostructures. The nomenclature of the PECA-coated samples follows a systematic approach based on solvent composition and water content. For samples polymerised in only *n*-hexane, the designation takes the form (*x*)H(*z*)p, where *x* represents the percentage of *n*-hexane (abbreviated as H), and *z* indicates the water content (WC) in ppm. For coatings performed in *n*-hexane/toluene mixtures, the designation expands to (*x*)H(*y*)T(*z*)p, where *x* and *y* represent the percentages of *n*-hexane and toluene (abbreviated as H and T, respectively), followed by *z* denoting the WC range. In both cases, the letter ‘p’ is appended to signify ppm of the water in the solvent system. For example, the sample synthesised in pure *n*-hexane at the lowest water content (30 ppm) will be denoted as 100H30p. Subsequent designation of a sample fabricated within the same WC range but with a *V*_Hex_ : *V*_Tol_ = 4 : 1 will be designated as 80H20T30p *etc.* Finally, the sample with the highest water content (120 ppm) and the highest toluene proportion (*V*_Hex_ : *V*_Tol_ = 1 : 4) is denoted as 20H80T120p. SEM top-view images revealed that the majority of the obtained PECA nanostructures (PECA-NS) are relatively similar to the alumina necklace-like nanostructures previously reported to be obtained *via* the DAGS mechanism in the gaseous phase.^12,13^ These PECA necklace-like nanostructures manifested in various forms, as prominent necklace-like nanostructures in form of either ‘continuous islands’ (Fig. 2b, n, r, and s) or ‘disrupted islands’ (Fig. 2g, o, and q). In Fig. 2a, d, e, h, i, and j the PECA necklace nanostructures still form in continuous islands but with a flatter surface profile. On the other hand, a few PECA-NS display punctual patterns on the surface (Fig. 2c and k), while others seem to build up a polymer film (Fig. 2m) with some sub-circular patterns as seen in Fig. 2f, l and t. SEM top-view images clearly indicate that minor modifications in the polymerisation parameters (solvent system and WC) can result in significant morphological variations within the PECA-NS. However, initial observations did not indicate a straightforward correlation between the PECA-NS morphology and the increase of either toluene fraction in *n*-hexane or WC values. This suggests that additional factors, including diffusion, adsorption of water molecules, and their stochastic distribution on the substrate, may interact to affect the development of PECA-NS *via* the DAGS mechanism in the solvent phase.

### Characterisation of PECA fibrous nanostructures (PECA-FNS)

#### SEM cross-sectional view of the PECA-NS

**Fig. 3:**
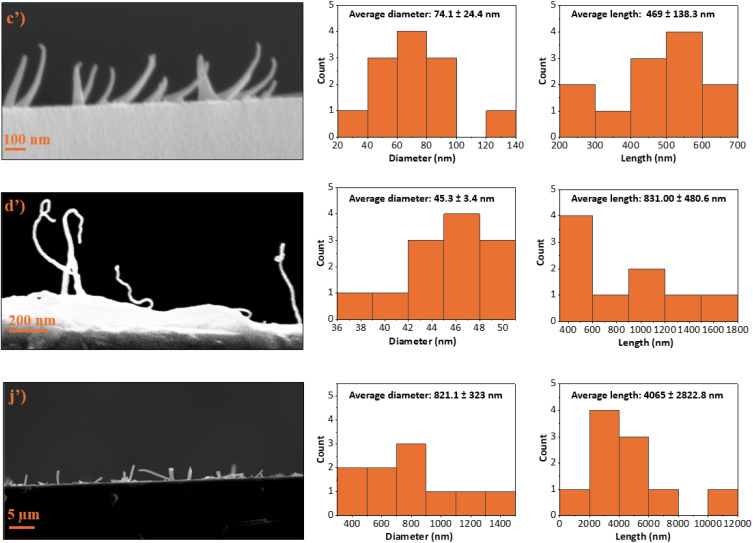
SEM side-view of the different PECA-FNS (on the left) and their corresponding diameter and length histograms (on the right starting from the top to the bottom): (c′) sample 60H40T30p, (d′) sample 40H60T30p, and (j′) sample 20H80T50p.

The SEM profile-view of samples 60H40T30p, 40H60T30p, and 20H80T50p revealed the presence of PECA fibrous nanostructures (PECA-FNS) as shown in Fig. 3c′, 3d′ and 3j′. The evaluation of the average length (*L*_av_) and average width (*d*_av_) of the observed PECA-FNS in sample 60H40T30p showed a *L*_av_ ≈ 469 nm and *d*_av_ ≈ 74.1 nm, while the PECA-FNS of sample 40H60T30p display a more elongated and thinner morphology, with an *L*_av_ ≈ 831 nm and a *d*_av_ ≈ 45.3 nm. Compared to these two previous samples, the FNS of sample 20H80T50p possess a significantly greater length and width (*L*_av_ ≈ 4065 nm and *d*_av_ ≈ 821.1 nm). The exact nature of the obtained PECA-FNS and their average aspect ratios (*L*_av_ : *d*_av_) are presented below in Table 1.

**Table 1:** The average aspect ratio of the different PECA-FNS

Sample	*L*_av_ : *d*_av_	Classification of the PECA-FNS
60H40T30p	6.4	Nanofibers (NF)
40H60T30p	18.3	Nanofibers (NF)
20H80T50p	4.9	Nanoribbons (NR)

**Fig. 4:**
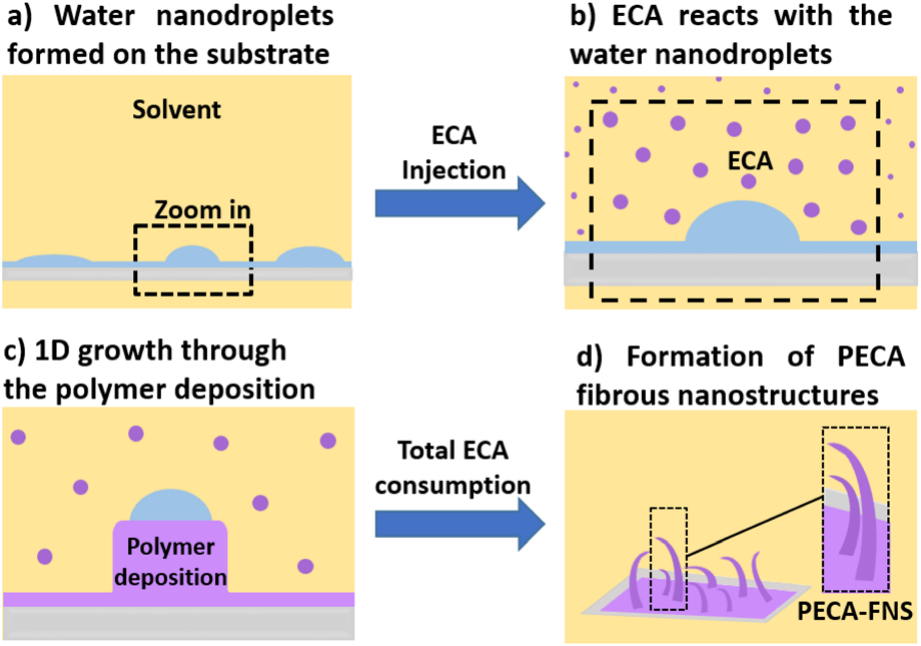
Illustration of the simplified DAGS mechanism during the growth of the PECA-FNS in the liquid phase: (a) the state of the water nanodroplets on the glass surface; (b) ECA molecules react with the water nanodroplets at the surface of the substrate; (c) the propagation of the polymerisation is ensured by the available ECA molecules and 1D growth is ensured by the PECA insolubility in water; (d) upon full consumption of ECA and or termination *via* water the PECA-FNS are formed on the substrate.

The observed aspect ratios of the obtained PECA-FNS are lower compared to those of previously fabricated silicone-based 1D nanostructures *via* the DAGS mechanism. For instance, silicone nanotubes with an aspect ratio up to 800 were produced in toluene with a water content of 155 ± 5 ppm,^24^ whereas in our case, the maximum water content used to polymerise ECA was 120 ppm. A plausible explanation for the dimensional differences between silicone-based and PECA-FNS may be attributed to the distinct polymerisation mechanisms of ECA and silanes. ECA undergoes polyaddition, characterised by the sequential addition of monomer molecules without the release of by-products. In this case, water acts as an initiator, and trace amounts are already sufficient to induce a spontaneous anionic polymerisation.^25^ Conversely, silanes polymerise *via* hydrolysis with a subsequent polycondensation. Polycondensation is a step-growth polymerisation that involves the elimination of small molecules such as HCl. In silane polymerisation, water functions more as a reactant, and the molar water-to-silane ratio is crucial in controlling the overall polymerisation, alongside other factors such as pH, temperature or catalysts.^26^ The contrasting role of water in each process – as an initiator in ECA polyaddition and as a reactant in silane polycondensation is likely to also contribute to the distinct dimensional variations in the corresponding polymeric 1D nanostructures obtained by means of the DAGS mechanism. While the precise conditions and factors contributing to the formation of PECA-FNS require further investigation, the morphological similarities of the PECA-FNS to the previously obtained 1D inorganic nanostructures *via* the DAGS mechanism still provides evidence for a shared underlying growth process as shown in Fig. 4. In addition to the hydroxyl (OH) groups present on the pre-treated glass substrate, we believe that water nanodroplets formed on its surface also serve as initiation sites for the ECA anionic polymerisation (Fig. 4a). Once initiated, the reaction continues propagating as long as ECA molecules are available to sustain chain growth of the macro-carbanion (Fig. 4b). The resulting PECA is then deposited due to its water insolubility,^27^ ensuring a one-dimensional growth of the PECA-FNS (Fig. 4c). This process continues until the full consumption of ECA and/or until the active PECA chain interacts with a nearby water molecule, which will terminate the polymerisation and lead to the formation of the PECA-FNS (Fig. 4d). Moreover, the solvent is thought to have a significant influence on the growth mechanism of the PECA-FNS, since the nature of the solvent determines the interactions polymer–solvent, which are known to impact the macromolecular conformation of the polymer chains.^28^ The interaction of a solute-which are here ECA, water, and the later formed PECA with a certain solvent, can be predicted *via* the Hansen solubility parameters (HSP). This approach is based on the principle ‘like dissolves like’ and divides cohesive energy into three components: dispersion interactions, polar interactions, and hydrogen bonding,^29–32^ which are represented by their respective energy densities *δ*_d_, *δ*_p_, and *δ*_H_ (MPa^1/2^ in the SI system).^33^ In general, if the HSP components (*δ*_d_, *δ*_p_, and *δ*_H_) of a solvent and a solute are close enough, the two substances are likely to be ‘compatible’.^30^ We aim to clarify the formation of the PECA-FNS in a simplified manner by utilising HSP, with a primary focus on the interactions between the solvent and water. We also assert that the interactions involving ECA with water, as well as those between ECA and the solvent system and the glass surface, likely play a significant role in the one-dimensional growth *via* the DAGS mechanism. The morphological differences observed in the PECA-FNS between samples 60H40T30p and 40H60T30p can be attributed to variations in solvent compositions, given that both samples were polymerised with the same amount of water. This modification suggests a change in how the solvent system interacts with water nanodroplets on the glass substrate. For example, in sample 60H40T30p, where *n*-hexane predominates, the substantial difference in HSP values between water and *n*-hexane (see Table 2) compels the formation of larger water nanodroplet ‘islands’ on the substrate, likely due to a greater affinity for the hydrophilic glass surface. This phenomenon probably leads to rapid initial consumption of the monomer, resulting in wider but shorter nanofibers due to the limited availability of ECA molecules necessary for supporting macromolecular chain propagation. In contrast, in sample 40H60T30p, where toluene is the predominant solvent, the favourable interaction between water and toluene, characterised by a minimal difference in HSP values, as evidenced in Table 2, facilitates the formation of smaller water islands on the glass surface. This distribution of smaller initiation sites contributes to the development of thinner nanofibers. Furthermore, the higher proportion of toluene in sample 40H60T30p likely retains more unreacted ECA molecules in solution, which can further support macromolecular growth after initiation, ultimately resulting in longer PECA nanofibers. On the other hand, the PECA-FNS observed in the sample 20H80T50p exhibit both a larger *d*_av_ and *L*_av_, likely due to wider and elongated water islands formed on the substrate surface, which could potentially explain their ribbon like appearance. Additionally, the solvent mixture in this sample, primarily composed of toluene, might have facilitated the interactions among all solutes (ECA, water and the growing PECA), promoting the propagation of significantly longer polymeric chains or even causing the arrangement of PECA macromolecules, resulting in a longer *L*_av_.

**Table 2:** Hansen solubility parameters (HSP) of ECA, *n*-hexane, toluene, and water in MPa^1/2^ (ref. 34)

	*δ*_d_	*δ*_p_	*δ*_H_
ECA	15.2	10.3	9
*n*-Hexane	14.9	0	0
Toluene	18	1.4	2
Water	15.5	16	42.3

#### FT IR measurement of the PECA-FNS

**Fig. 5:**
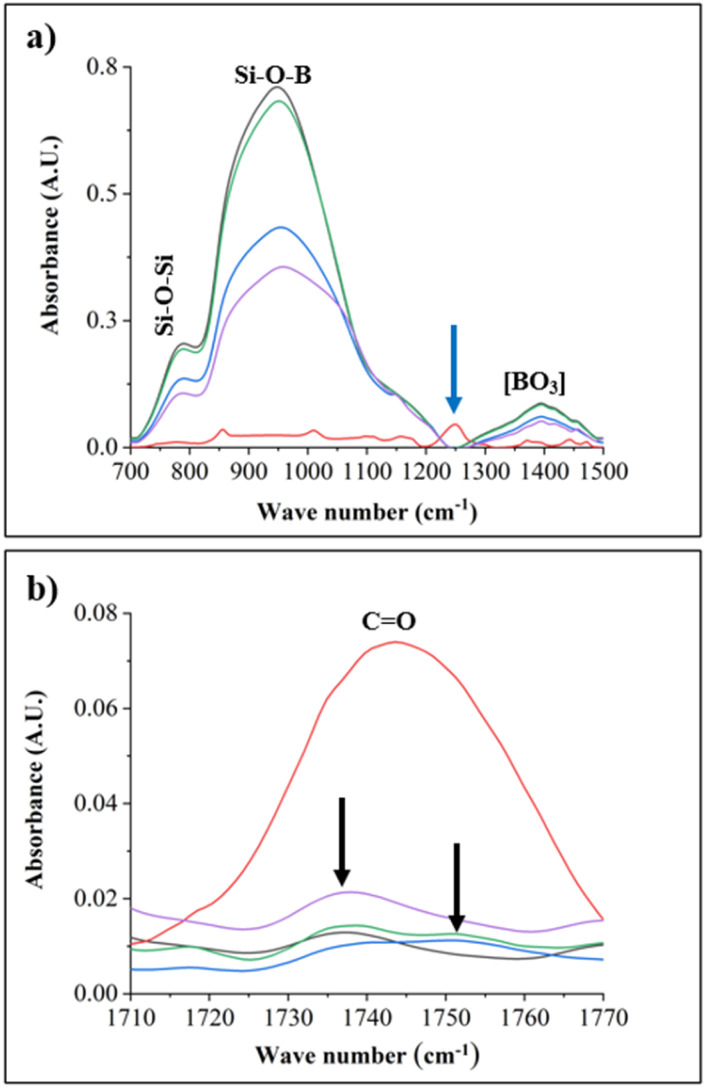
FT-IR spectra of crude PECA (red), 60H40T30p (blue); sample 40H60T30p (green) sample 20H80t50p (purple); and a pristine pre-treated glass slide (black). (a) Highlights prominent absorption spectra on the glass substrates despite the PECA-FNS coating, while (b) displays the C=O stretching band indicative of the PECA polymer.

The presence of PECA-FNS was further confirmed by ATR FT-IR spectra, which were compared to the IR spectra of a clean pristine glass slide and crude PECA (Fig. 5). The latter was prepared by dropping 0.85 mmol on a pre-treated glass slide and polymerised at ambient temperature (*T* = 21 ± 1 °C) and an ambient relative humidity RH ≈ 52%.

It is noticeable that the signals from glass are very prominent in the PECA-coated substrates as shown in Fig. 5a.

This might be due to the low concentration of ECA used during polymerisation. The peak at 783 cm^−1^ is attributed to the symmetric stretching of Si–O–Si,^35^ while the sharp band at 956 cm^−1^ corresponds to the stretching of the Si–O–B band.^36^ The blue arrow around 1250 cm^−1^ shows the absorption of the C–O–C bond coming from the crude PECA.^37^ In contrast, this absorption band was blended by signals from the glass substrate in all the PECA-FNS samples. The broad band at 1395 cm^−1^ represents the asymmetric stretching relaxation of the B–O bond originating from the trigonal BO_3_ units of glass.^38^ Finally, the IR signals at ∼1738–1753 cm^−1^ (Fig. 5b) can be ascribed to the carbonyl groups found in PECA. The black arrows indicate a shift in the C=O bond of the PECA-FNS samples compared to the C=O absorption band observed in the sample with crude PECA.

#### Topological properties of the PECA-FNS

**Fig. 6:**
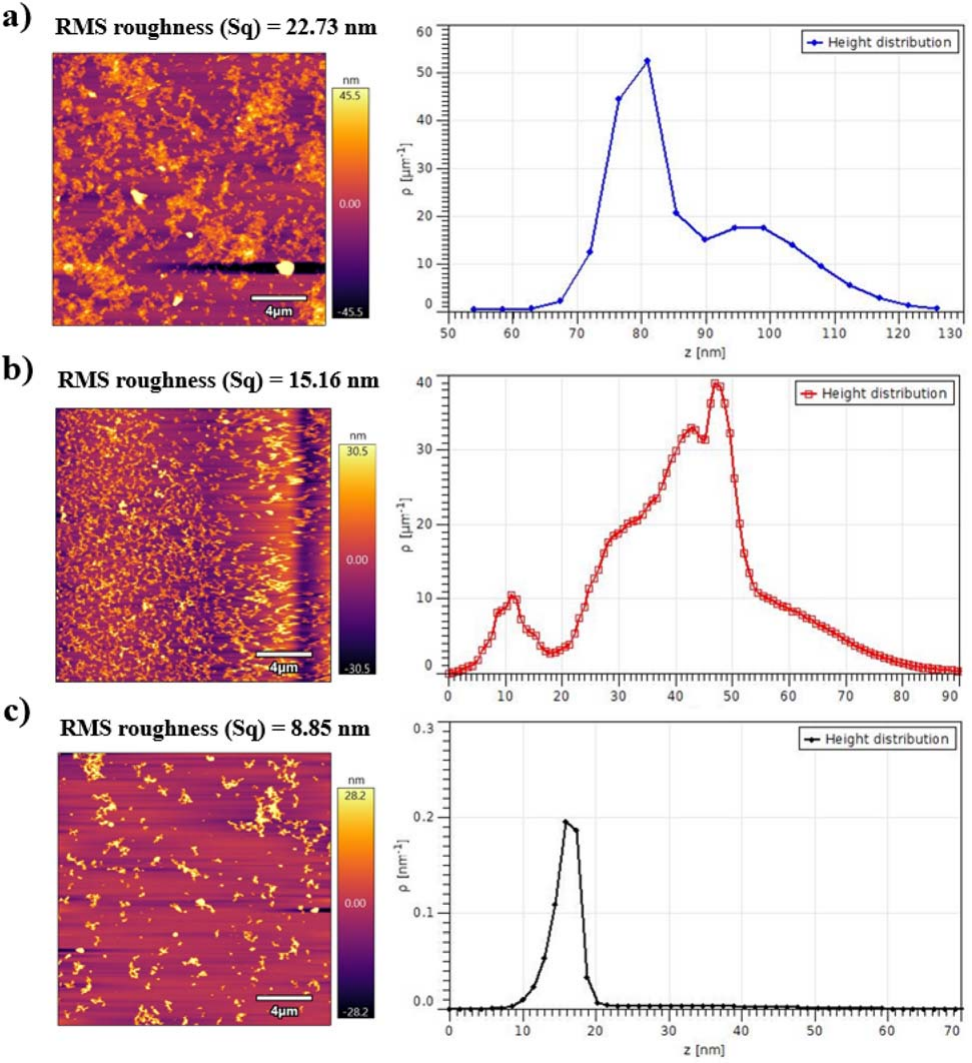
AFM top-view images (20 μm × 20 μm) (on the right) and their corresponding height distribution histogram represented by the surface features *ρ* (μm^−1^) against the height *z* (nm): (a) sample 60H40T30p, (b) sample 40H60T30, and (c) sample 20H80T50p.

Atomic force microscopy (AFM) analysis was conducted on the PECA-FNS surfaces to investigate their surface morphology and topographical features. Fig. 6 presents the AFM top-view images and corresponding height distributions, providing crucial insights into the surface of the PECA-FNS.

The background of sample 60H40T30p shows a rough surface with a root mean square roughness (RMS roughness Sq) of 22.73 nm, and a bimodal height distribution characterised by two distinct *ρ* peaks. The primary peak occurred at ∼53 μm^−1^, corresponding to a height of approximately 81 nm, while the secondary peak reached a minimum of ∼18 μm^−1^ at a height of circa 97 nm. This bimodal distribution suggests a hierarchical surface structure with two predominant PECA-NFs populations with distinct different heights. In contrast, sample 40H60T30p displayed a smoother surface (Sq = 15.16 nm) with a multimodal height distribution, indicative of a heterogeneous surface topography. The maximum peak was observed at ∼38 μm^−1^, corresponding to a height of approximately 47 nm, while the minimal peak appears at ∼10 μm^−1^ for a height of *ca.* 10 nm. This complex distribution implies a highly varied surface topography, potentially comprising both small-scale rough and other larger PECA-NFs. The surface of sample 20H80T50p was the smoothest (Sq = 8.85 nm). Its height distribution was relatively narrow and symmetric with a *ρ* value of 0.19 μm^−1^, corresponding to a maximum height of *ca.* 15 nm. This uniform height distribution suggests a homogeneous surface topography with consistent PECA-NRs morphologies.

#### Optical properties of the PECA-FNS

**Fig. 7:**
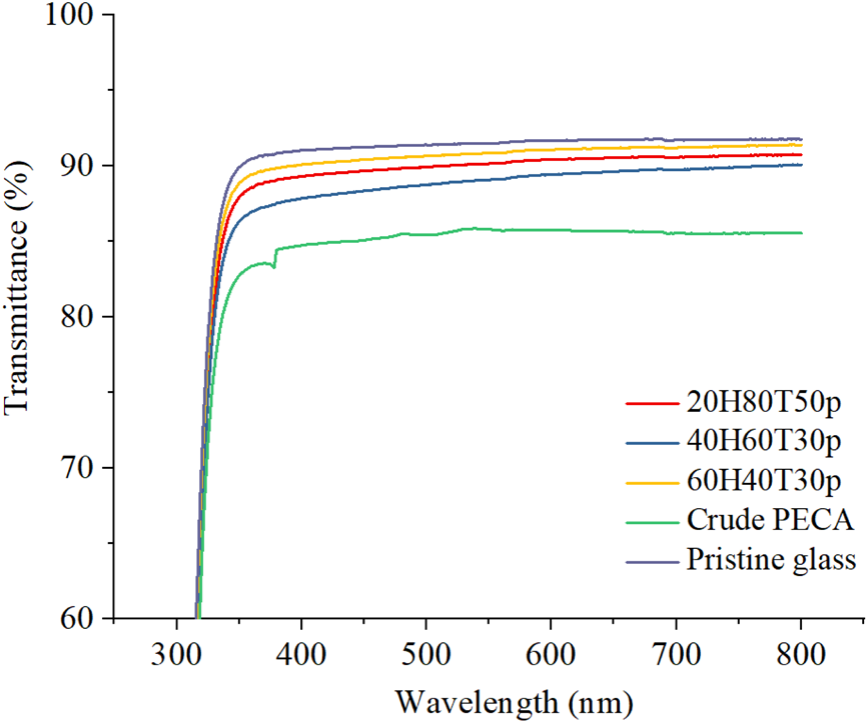
Transmittance spectra measured from 300–800 nm: crude PECA (green), sample 60H40T30p (yellow), sample 40H60T30p (blue), 80H20T50p (red), and a pristine pre-treated glass slide (purple).

Transmittance measurements between 300 and 800 nm were performed to evaluate the transparency of samples coated with PECA-FNS. Two reference samples were used for comparison: a pre-treated, pristine glass slide and a glass slide coated with crude PECA as displayed in Fig. 7. The pristine glass exhibited the highest maximum transmittance of 91.80%, while the one coated with crude PECA showed the lowest value of 85.57%. The transmittance of the PECA-FNS samples fell between these two reference values, reaching maximum transmittance levels of 91.41% for sample 60H40T30p, 90.77% for sample 20H80T50p, and 90.10% for sample 40H60T30p. The transparency of PECA-FNS coatings can be comparable to^39^ that of glass, primarily because PECA is an amorphous polymer. In amorphous polymers, the molecular chains are arranged randomly, which facilitates the passage of light with minimal scattering. In contrast, semi-crystalline polymers contain both ordered crystalline and amorphous areas. These crystalline domains can serve as scattering centres, leading to the diffraction of incident light and consequently reducing overall transparency. As a result, amorphous polymers typically exhibit greater transparency than their semi-crystalline counterparts.^40,41^

The differences in transmittance noted within the PECA-FNS samples can be attributed to variations in their topology or coating thickness. These discrepancies can lead to light scattering, resulting in reduced light transmission. For example, sample 40H60T30p exhibits the lowest transmittance value among all the PECA-FNS samples, likely due to optical losses associated with its heterogeneous surface, as indicated by previous AFM topological analysis. However, the overall high transparency of the PECA-FNS can be well suited for coating applications, in which preserving the original appearance of the substrate (*i.e.*, colour) is required. When compared to the previously fabricated silicone nanofilaments, the transmittance values of the PECA-FNS in the visible range remain slightly lower.^42^

#### Wettability of the PECA-FNS

**Fig. 8:**
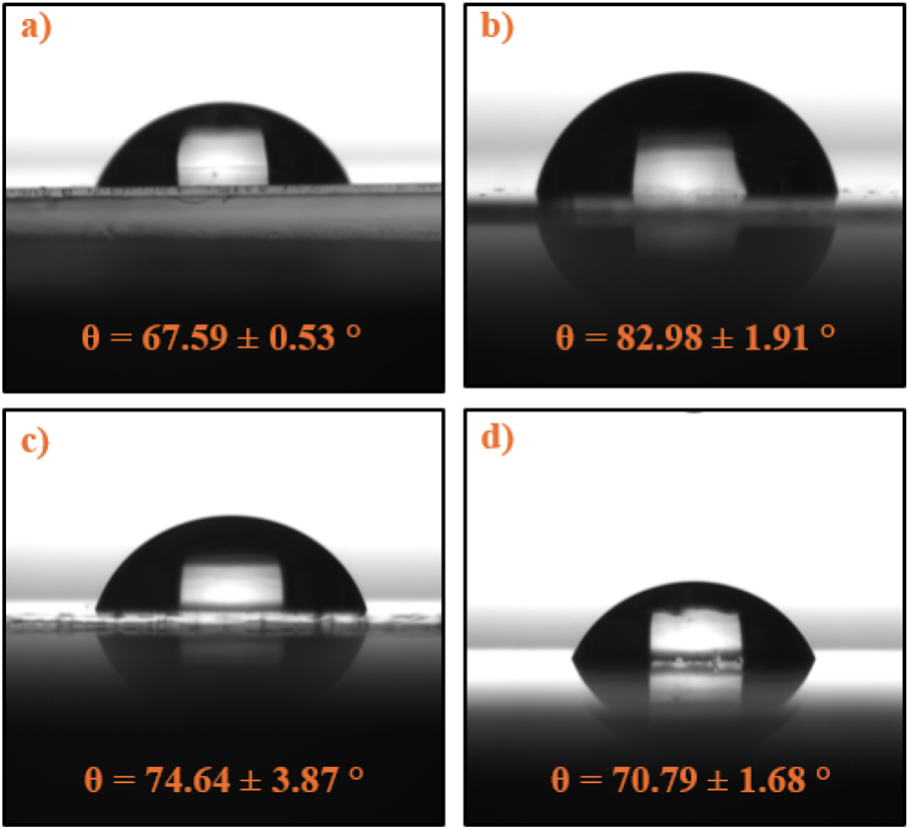
Static contact angle measurements on PECA-coated glass substrates: (a) crude PECA on glass, (b) sample 60H40T, (c) sample 40H60T30p, and (d) sample 20H80T50p.

The wettability of the PECA-FNS samples was assessed through static contact angle measurements, revealing a range of moderate hydrophilicity across all specimens as displayed in Fig. 8.

Contact angles varied from 67.59° for crude PECA on glass (Fig. 8a) to 82.98° for sample 60H40T30p (Fig. 8b), with samples 40H60T30p and 20H80T50p exhibiting intermediate values of 74.64° (Fig. 8c) and 70.79°(Fig. 8d), respectively. These results indicate a slight enhancement in hydrophobicity compared to the crude PECA coating, although the surfaces can not be described as hydrophobic, as the contact angle does not exceed 90°.^43^ The observed moderate hydrophilicity of PECA-FNS aligns with expectations based on its molecular structure, which contains polar CN and COOEt groups. Previous studies have estimated the surface tension of PECA at 33 mN m^−1^,^44^ and our findings for the contact angle of the crude PECA are consistent with reported values in the literature.^45,46^ Despite the complex relationship between surface roughness and wettability,^47^ a correlation between surface roughness (Sq) and contact angle was observed, in our case: the contact angle increased with increasing Sq, suggesting that topographical features of the PECA-FNS have a role in modulating surface wettability. Interestingly, sample 60H40T30p exhibited the highest hydrophobicity among all the measured samples, which may be attributed to its high surface roughness (Sq = 22.73 nm). It is also noteworthy that PECA-FNS samples displayed markedly different wetting behaviour compared to previously reported PECA nanofibers (CA = 158°).^46^ However, the moderately hydrophilic nature of the PECA-FNS could be advantageous for example in wound healing applications where a balance between hydrophobicity and hydrophilicity is desired.^48^

## Conclusions

In this study, we successfully demonstrated the extension of the DAGS mechanism to organic monomers using ECA. The anionic polymerisation was carried out in *n*-hexane and *n*-hexane/toluene mixtures with water contents ranging from 30 to 120 ppm, resulting in PECA fibrous nanostructures (PECA-FNS) appearing as either nanofibers (PECA-NFs) and or nanoribbons (PECA-NRs). The aspect ratio of the PECA-FNS ranges from 4.9 to 18.3. These 1D nanostructures bear resemblance to the well-known silicon nanofilaments (SNF) formed *via* the DAGS mechanism, providing evidence that a similar mechanism underpins the growth of these organic nanostructures. This similarity further validates the versatility of the DAGS mechanism and highlights its potential for producing a wide range of one-dimensional nanomaterials. While the exact factors contributing to the formation of the PECA-FNS remain partially unrevealed, the applicability of the DAGS mechanism to organic monomers represents a significant advancement, as it enables the fabrication of organic 1D nanostructures while retaining the advantages of DAGS, including operation under mild conditions at room temperature and atmospheric pressure. This extension not only broadens the scope of the mechanism but also opens new opportunities for developing hybrid materials with tuneable and synergistic properties, significantly expanding the application potential of 1D polymeric nanostructures. Notably, PECA-FNS exhibit relatively high transparency and moderate hydrophilicity, positioning them as a promising alternative to nanocoatings prepared *via* the DAGS mechanism, particularly when both tailored surface wettability and excellent transparency are desired.

## Conflicts of interest

There are no conflicts to declare.

## Data Availability

All the raw data used in this article can be accessed from OSF repository at OSF|PECA-FNS *via* the DAGS mechanism (https://osf.io/mkd7p/).
